# Gender differences in response and remission rates among patients receiving esketamine

**DOI:** 10.1192/j.eurpsy.2023.385

**Published:** 2023-07-19

**Authors:** A. T. Guterman, K. Feffer, A. Z. Gershi

**Affiliations:** Lev-Hasharon Mental Health Center, Tzur Moshe, Israel

## Abstract

**Introduction:**

Depressive disorders are among the leading causes of disability. The lifetime risk for developing clinically significant depression is estimated around 15%. Multiple studies have found female-male ratio of 2:1 across nations and cultures. The reason for this marked difference is still not clear, although several theories addressing social, biological, and environmental contributors have been suggested. Respond to anti-depressant treatment is considerably similar in both genders. However, several studies have shown worse outcomes among women during menopause.

In 2019, the US Food and Drug Administration (FDA) approved a nasal spray formulation of esketamine for the treatment of resistant depression (TRD) in adults, and the treatment has been approved to use by the ministry of health in Israel since 2020 . Recent studies found more than 50% response and 35% remission rates, when nasal esketamine was given as an add-on therapy.

**Objectives:**

to assess gender differences in response and remission rates among patients receiving esketamine as an add-on therapy for TRD in a real-world setting

**Methods:**

23 female and 22 males received at least 12 weeks of esketamine treatment in a day-care setting. Data collected included depression, dissociation and quality of life scales.

**Results:**

Females tend to experience more severe dissociation during treatment. Average response and remission rates were similar in both groups (48% and 32% respectively), however females were less favorable to show rapid response (during the first 4 weeks). among responders no major differences in time to response has been found.

**Conclusions:**

no major gender differences in response or remission rates have been found among patients receiving esketamine treatment for TRD aside from lower rates of rapid response. Additionally, Females report more severe dissociation during treatment.

**Disclosure of Interest:**

None Declared

